# Predicting microbe-disease associations via graph neural network and contrastive learning

**DOI:** 10.3389/fmicb.2024.1483983

**Published:** 2024-12-13

**Authors:** Cong Jiang, Junxuan Feng, Bingshen Shan, Qiyue Chen, Jian Yang, Gang Wang, Xiaogang Peng, Xiaozheng Li

**Affiliations:** ^1^College of Computer Science and Software Engineering, Shenzhen University, Shenzhen, China; ^2^National Engineering Laboratory for Big Data System Computing Technology, Shenzhen University, Shenzhen, China; ^3^College of Management, Shenzhen University, Shenzhen, China; ^4^Beijing Key Laboratory of Mental Disorders, National Clinical Research Center for Mental Disorders and National Center for Mental Disorders, Beijing Anding Hospital, Capital Medical University, Beijing, China; ^5^Advanced Innovation Center for Human Brain Protection, Capital Medical University, Beijing, China; ^6^College of Life Sciences and Oceanography, Shenzhen University, Shenzhen, China; ^7^JCY Biotech Ltd., Pingshan Translational Medicine Center, Shenzhen Bay Laboratory, Shenzhen, China

**Keywords:** microbe-disease associations, graph convolutional network, graph attention mechanism, contrastive learning, gut microbial metagenomics

## Abstract

In the contemporary field of life sciences, researchers have gradually recognized the critical role of microbes in maintaining human health. However, traditional biological experimental methods for validating the association between microbes and diseases are both time-consuming and costly. Therefore, developing effective computational methods to predict potential associations between microbes and diseases is an important and urgent task. In this study, we propose a novel computational framework, called GCATCMDA, for forecasting potential associations between microbes and diseases. Firstly, we construct Gaussian kernel similarity networks for microbes and diseases using known microbe-disease association data. Then, we design a feature encoder that combines graph convolutional network and graph attention mechanism to learn the node features of networks, and propose a feature dual-fusion module to effectively integrate node features from each layer's output. Next, we apply the feature encoder separately to the microbe similarity network, disease similarity network, and microbe-disease association network, and enhance the consistency of features for the same nodes across different association networks through contrastive learning. Finally, we pass the microbe and disease features into an inner product decoder to obtain the association scores between them. Experimental results demonstrate that the GCATCMDA model achieves superior predictive performance compared to previous methods. Furthermore, case studies confirm that GCATCMDA is an effective tool for predicting microbe-disease associations in real situations.

## 1 Introduction

Microbes are primarily composed of bacteria, fungi, archaea, and viruses, predominantly inhabit the gut within the human body (Sommer and Bäckhed, 2013; Blum, 2017). The gut microbiota is closely associated with human health, playing a crucial role in regulating host physiological processes, such as immunity and metabolism (Lynch and Pedersen, 2016; Tooley, 2020). In recent years, biological experiments have demonstrated that dysbiosis or imbalance in the human microbiota could cause human diseases (Marchesi et al., 2016), such as liver diseases (Henao-Mejia et al., 2013), diabetes (Paun et al., 2017), obesity (Tseng and Wu, 2019), and even cancer (Schwabe and Jobin, 2013). However, traditional biological experiments suffer from drawbacks such as long experimental cycles and expensive costs. Therefore, if we can utilize effective computational methods to predict potential sets of associations between microbes and diseases in advance, it would be possible to reduce unnecessary experimental trials and costs in traditional biological experiments, thereby accelerating the development of research in the field of microbe-disease associations.

Current computational methods for predicting microbe-disease associations can primarily be categorized into three categories, namely network-based methods, random walk-based methods, and deep learning-based methods. The network-based methods infer the potential association between microbes and diseases by utilizing the topological information within the network. For example, Chen et al. (2017) proposed a KATZHMDA model based on the KATZ measure, which scores potential disease related microbes by calculating all paths of different lengths between microbes and diseases. Bao et al. (2017) proposed the Network Consistency Projection for Human Microbe-Disease Association Prediction (NCPHMDA) model, evaluating the association scores between microbes and diseases by computing disease space projection scores and microbe space projection scores. Long and Luo (2019) designed a meta-graph-based method named WMGHMDA, which calculates the probability scores of microbe-disease pairs by utilizing a weighted meta-graph search algorithm on a heterogeneous network. Wang et al. (2023) proposed a SAELGMDA model by combining sparse autoencoder and Light Gradient boosting machine.

The success of random walk algorithms in graph data processing has prompted researchers to propose various microbe-disease association prediction algorithms based on this approach. For instance, Zou et al. (2017) developed a novel computational model of BiRWHMDA, which predicts potential microbe-disease associations by bi-random walks on a heterogeneous network. Luo and Long (2018) proposed a novel computational model of NTSHMDA, which integrates network topology similarity into the restarted random walk algorithm to distinguish the walking probabilities of disease-microbe node pairs. Yan et al. (2019) introduced a BRWMDA method, predicting potential microbe-disease associations by executing bi-random walks with different steps on microbe and disease networks.

With the significant achievements of deep learning algorithms in various research fields, researchers have gradually begun to explore the application of these algorithms in the task of predicting the associations between microbes and diseases. For example, Ma and Jiang (2020) developed an end-to-end graph convolutional neural network-based mining model NinimHMDA to predict different types of microbe-disease associations. Long et al. (2021) proposed a novel deep learning framework of GATMDA, which utilizes graph attention networks along with inductive matrix completion for predicting human microbe-disease associations. Hua et al. (2022) developed a multi-view graph augmentation convolutional network (MVGCNMDA) to predict potential disease-associated microbes. Jiang et al. (2022) proposed the KGNMDA method, using a knowledge graph neural network method for predicting microbe-disease associations. Peng et al. (2023) developed a computational method for predicting microbe-disease associations, named GPUDMDA, which integrates graph attention autoencoder, positive-unlabeled learning, and deep neural network.

In addition to the three mainstream methods mentioned, some computational approaches for microbe-disease prediction have been developed based on regularization and matrix factorization/completion techniques. For instance, Wang et al. (2017) proposed a semi-supervised computational model of Laplacian Regularized Least Squares for Human Microbe—Disease Association (LRLSHMDA) to predict microbe-disease associations. Shen et al. (2017) developed a computational method of CMFHMDA, which utilizes collaborative matrix factorization to reconstruct correlation matrices between diseases and microbes. Liu et al. (2023) proposed a novel method called MNNMDA to predict microbe-disease associations by applying a Matrix Nuclear Norm method.

Among the methods mentioned above, network-based and random walk-based methods may encounter constraints in learning features of nodes representing microbes and diseases with few known associations, due to the limited information propagation caused by the sparsity of the microbe-disease association network. Meanwhile, matrix factorization/completion methods can only capture linear associations, thus failing to accurately capture the nonlinear interactions between microbes and diseases. Recent studies have suggested that graph neural network algorithms in deep learning could offer a more effective approach for learning node features in microbe-disease association networks. Therefore, this study further attempts to design node feature learning algorithms based on graph neural networks, aiming to obtain more effective node features from the microbe-disease association network, thereby predicting more accurate candidate sets of microbe-disease associations.

In this work, we propose a deep learning framework named GCATCMDA, which explores the application of graph neural networks for the microbe-disease association prediction task. First Gaussian kernel similarity is calculated based on known microbe-disease association data to construct microbe similarity networks and disease similarity networks. We then combine graph convolutional networks and graph attention mechanisms to learn feature representations of microbes and diseases in different networks, and propose a feature dual-fusion module to effectively integrate node features generated by each graph attention layer. Next, we utilize contrastive learning to enhance the feature consistency of the same microbe (or disease) across different association networks. Finally, the obtained microbe and disease features are inputted into an inner product decoder to compute their corresponding association scores. The model can obtain better node features through GCAT aggregation. In addition, contrastive learning increases the distance between nodes, allowing the model to better distinguish nodes and make subsequent predictions better. Experimental results demonstrate that the GCATCMDA model achieves better predictive performance compared to previous methods, and case studies of obesity and IBD (inflammatory bowel disease) confirm the high accuracy of the microbe-disease association candidate set produced by our method.

## 2 Materials and methods

### 2.1 Datasets

The dataset in this study was sourced from the HMDAD database (http://www.cuilab.cn/hmdad), which collects known associations between microbes and diseases by searching past research literature (Ma et al., 2017). HMDAD adapted a systematic approach by only including associations that have been experimentally validated and published in reputable journals. This ensures a high level of reliability in the dataset. Past researchers commonly employ metagenomic sequencing techniques to analyze fluctuations in microbial community abundance within specific diseases, contrasting them with the microbial compositions of healthy individuals, thus exploring the associations between microbes and diseases. In the HMDAD dataset, a microbe-disease association pair may contain multiple entries from different research literature sources. Therefore, here, we regard the same microbe-disease association from different evidences as a pair, further removing the redundant information present in the HMDAD dataset. Finally, for this study, we employed a dataset consisting of 450 microbe-disease associations, encompassing 39 human diseases and 292 microbes.

### 2.2 Problem definition

For the convenience of clarity in describing the subsequent research methods, we provide a simple problem definition for the task of predicting associations between microbes and diseases here. We denote *M* = {*m*_1_, *m*_2_, …, *m*_*n*_*m*__} and *D* = {*d*_1_, *d*_2_, …, *d*_*n*_*d*__} as the sets representing *n*_*m*_ microbes and *n*_*d*_ diseases, respectively. The matrix $A\in {\mathbb{R}}^{n_{m}\times n_{d}}$ represents the known associations between microbes and diseases, where *A*_*ij*_ = 1 if microbe *m*_*i*_ is associated with disease *d*_*j*_, otherwise *A*_*ij*_ = 0. However, *A*_*ij*_ = 0 does not mean that microbe *m*_*i*_ has no relation with disease *d*_*j*_. It may be the reason that their association has not yet been discovered. Therefore, the task of predicting associations between microbes and diseases aims to find microbe *m*_*i*_ for each disease *d*_*j*_ where *A*_*ij*_ = 0 in the known association matrix, but microbe *m*_*i*_ is actually related to disease *d*_*j*_.

### 2.3 GCATCMDA

**Figure 1:**
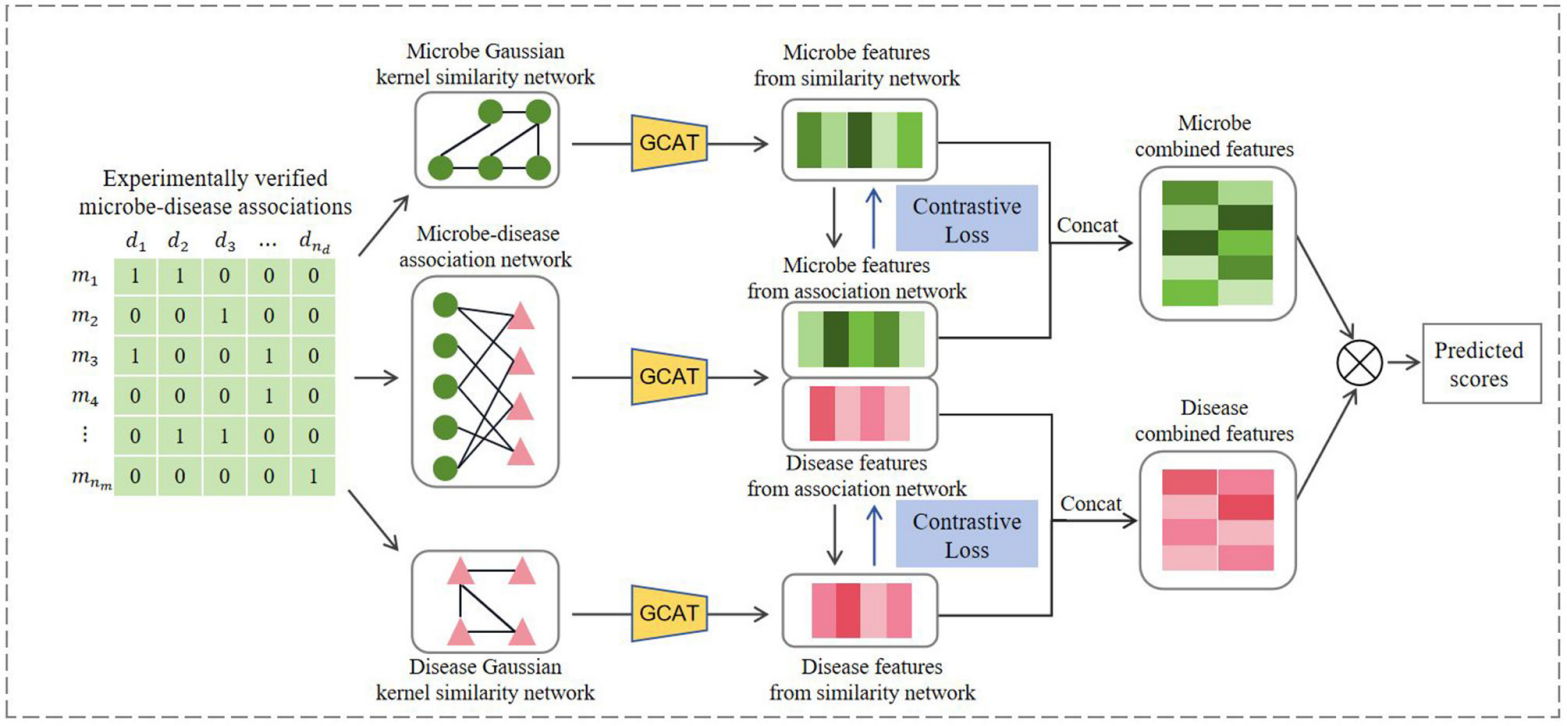
The workflow of GCATCMDA for microbe–disease prediction.

Figure 1 illustrates the workflow of GCATCMDA, a model based on graph neural networks and contrastive learning for predicting effective candidate sets of microbe-disease associations. First microbe-microbe and disease-disease Gaussian kernel similarity networks are constructed using known associations. The model then integrates graph neural networks and contrastive learning principles to extract meaningful feature representations of microbes and diseases from the association networks. Last the obtained microbe and disease features are fed into an inner product decoder to compute their corresponding association scores. A detailed description of the key components of this model is elucidated below.

#### 2.3.1 Microbe and disease similarity network construction

Previous study (Chen et al., 2017) have hypothesized that functionally similar microbes (or diseases) tend to exhibit similar interaction or non-interaction patterns with similar diseases (or microbes). They utilize Gaussian kernel functions to measure the similarity between two microbes (or diseases) in the same space. Therefore, in this study, we consider constructing microbe and disease similarity networks based on Gaussian kernel similarity scores for microbes and diseases.

We have recorded the known associations between microbes and diseases using the association matrix $A\in {\mathbb{R}}^{n_{m}\times n_{d}}$. The calculation formulas for the Gaussian kernel similarity score between microbe *m*_*i*_ and *m*_*j*_, and between disease *d*_*i*_ and *d*_*j*_, are as follows:


(1) $$\begin{array}{l} \text{KM}\left(m_{i},m_{j}\right)=\exp\left(-\lambda_{m}||\text{IP}\left(m_{i}\right)-\text{IP}\left(m_{j}\right)|{|}^{2}\right) \end{array}$$

(2) $$\begin{array}{l} \text{KD}\left(d_{i},d_{j}\right)=\exp\left(-\lambda_{d}||\text{IP}\left(d_{i}\right)-\text{IP}\left(d_{j}\right)|{|}^{2}\right) \end{array}$$

where *KM*(*m*_*i*_, *m*_*j*_) represents the Gaussian kernel similarity score between microbes *m*_*i*_ and *m*_*j*_, and *KD*(*d*_*i*_, *d*_*j*_) represents the Gaussian kernel similarity score between diseases *d*_*i*_ and *d*_*j*_. The term IP(*m*_*i*_) represents the *i*-th row of the association matrix *A* recording the associations between microbe *m*_*i*_ and other diseases, IP(*d*_*i*_) represents the *i*-th column of the association matrix *A* recording the associations between disease *d*_*i*_ and other microbes. The parameters λ_*m*_ and λ_*d*_ represent the normalized kernel bandwidths and are defined as follows:


(3) $$\begin{array}{l} \lambda_{m}=\frac{\lambda_{m}^{\prime }}{\frac{1}{n_{m}}\sum_{i=1}^{n_{m}}||\text{IP}\left(m_{i}\right)||} \end{array}$$

(4) $$\begin{array}{l} \lambda_{d}=\frac{\lambda_{d}^{\prime }}{\frac{1}{n_{d}}\sum_{i=1}^{n_{d}}||\text{IP}\left(d_{i}\right)||} \end{array}$$

where *n*_*m*_ and *n*_*d*_ represented the number of microbes and diseases. And $\lambda_{m}^{\prime }$ and $\lambda_{d}^{\prime }$ are the original bandwidths, and generally both set to 1.

We consider microbes (or diseases) to be strongly associated with each other when the Gaussian kernel similarity score between microbes (or diseases) exceeds a threshold of *t*. Therefore, the association matrices *MA* for microbes and *DA* for diseases can be expressed as follows:


(5) $$\begin{array}{l} \text{MA}\left(m_{i},m_{j}\right)=\{\begin{array}{cc} 1, & \text{ if KM }\left(m_{i},m_{j}\right)\geq t\\ 0, & \text{otherwise} \end{array} \end{array}$$

(6) $$\begin{array}{l} \text{DA}\left(m_{i},m_{j}\right)=\{\begin{array}{cc} 1, & \text{ if KD }\left(d_{i},d_{j}\right)\geq t\\ 0, & \text{otherwise} \end{array} \end{array}$$

#### 2.3.2 GCAT

**Figure 2:**
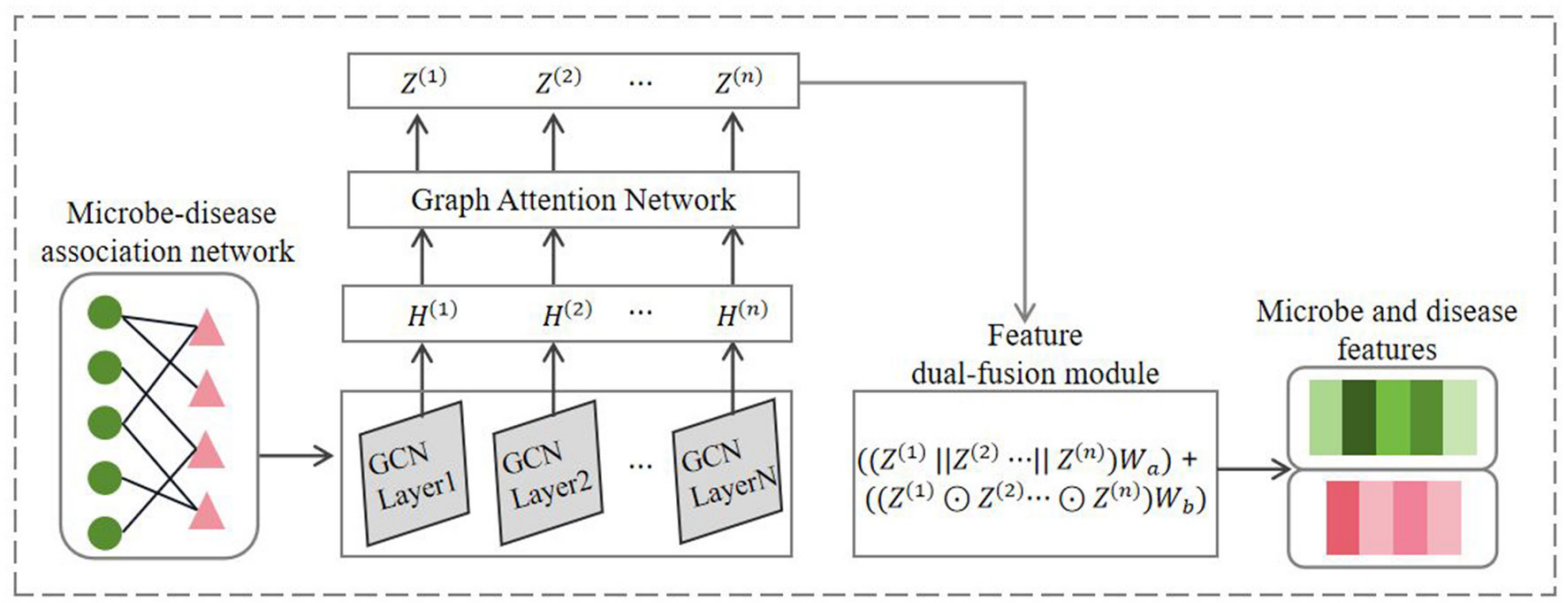
The flowchart of GCAT node feature encoding in microbe-disease association network.

Inspired by the work of Sun et al. (2022) on predicting metabolite-disease associations, this study adopted the GCAT feature encoder. The encoder initially combines graph convolution algorithms and graph attention mechanisms to learn the nodal features of the network, followed by the design of a feature dual-fusion module to effectively integrate the node features outputted by each graph attention layer. Since the GCAT feature encoder learns embedding representations on different association networks in a similar process, we take microbe-disease association network as an example to introduce the process of learning node features, as illustrated in Figure 2.

We represent the microbe-disease association network using a symmetric adjacency matrix $G\in {\mathbb{R}}^{\left(n_{m}+n_{d}\right)\times \left(n_{m}+n_{d}\right)}$, where *n*_*m*_ and *n*_*d*_ denote the numbers of microbes and diseases, respectively. The initial features of nodes in the network are represented by the matrix *H*^(0)^.


(7) $$\begin{array}{l} G=\left[\begin{array}{cc} 0 & A\\ A^{T} & 0 \end{array}\right] \end{array}$$

(8) $$\begin{array}{l} H^{\left(0\right)}=\left[\begin{array}{cc} KM & 0\\ 0 & KD \end{array}\right] \end{array}$$

Considering the ability of graph convolutional networks in capturing the global graph structural information, and the ability of graph attention mechanisms to assign attention weights to different nodes based on the local graph structure, the GCAT feature encoder integrates these two algorithms to effectively learn the node features of the microbe-disease association network. Firstly, the GCAT feature encoder linearly projects the node features of *H*^(0)^ onto a feature space of dimensional size *F*, denoted as *H*^(0)^ = *H*^(0)^*W*, where $W\in {\mathbb{R}}^{\left(n_{m}+n_{d}\right)\times F}$ is the weight matrix. Next, this module employs the graph convolutional networks (GCN) proposed by Kipf and Welling (2016) to learn node features in the network. GCN learns low-dimensional representations of nodes by aggregating neighbor node information through graph convolution operations while preserving the graph's structural information. The first-layer graph convolutional propagation formula for graph *G* can be expressed as:


(9) $$\begin{array}{l} {\text{H}}^{\left(1\right)}=\sigma \left({\tilde{D}}^{-\frac{1}{2}}\tilde{G}{\tilde{D}}^{-\frac{1}{2}}{\text{H}}^{\left(0\right)}{\text{W}}^{\left(0\right)}\right) \end{array}$$

Here, σ(.) denotes the activation function, $\tilde{G}=G+I$ represents the adjacency matrix with self-loops added, $\tilde{D}$ is the degree matrix of $\tilde{G}$, **W**^(0)^ denotes the trainable weight matrix of the first-layer graph convolution, and **H**^(1)^ represents the feature matrix outputted by the first-layer graph convolution.

Subsequently, the GCAT feature encoder enhances the learned node feature representations from the graph convolutional layers by incorporating a graph attention mechanism to aggregate weighted sums of neighbor information. In this study, we adopt the graph attention network (GAT) proposed by Veličković et al. (2017), which introduces an attention mechanism to assign different attention weights to the features of different neighbor nodes, enabling to focus on important neighbor features during aggregation for the target node. Thus, following the computation of the first-layer graph convolution, the attention scores $\alpha_{ij}^{\left(1\right)}$ for node *j* with respect to its neighbor node *i* in graph *G* can be calculated as:


(10) $$\begin{array}{l} \alpha_{ij}^{\left(1\right)}=\frac{\exp\left(f\left(\left[W_{\text{att}}^{\left(1\right)}h_{i}^{\left(1\right)}||W_{\text{att}}^{\left(1\right)}h_{j}^{\left(1\right)}\right]\right)\right)}{\sum_{k\in N_{i}}\exp\left(f\left(\left[W_{\text{att}}^{\left(1\right)}h_{i}^{\left(1\right)}||W_{\text{att}}^{\left(1\right)}h_{k}^{\left(1\right)}\right]\right)\right)} \end{array}$$

where || denotes the concatenation operation, $h_{*}^{\left(1\right)}$ represents the node features obtained by the graph *G* through the first-layer graph convolution, $W_{\text{att}}^{\left(1\right)}$ represents the weight matrix for the linear transformation of node features, *N*_*i*_ denotes the first-order neighboring nodes of node *i*. The attention mechanism *f*(·) is a single-layer feedforward neural network, parametrized by a weight vector $\vec{a}\in {\mathbb{R}}^{2F}$, and applying the LeakyReLU nonlinearity. We further employs a multi-head attention mechanism to stabilize the process of learning node representations in attention networks. It aggregates the features obtained from all attention heads by taking their average. Thus, the updated feature $z_{i}^{\left(1\right)}$ for node *i* via graph attention mechanism can be expressed as follows:


(11) $$\begin{array}{l} z_{i}^{\left(1\right)}=\sigma \left(\frac{1}{K}\overset{K}{\underset{k=1}{\sum }}\underset{j\in N_{i}}{\sum }\alpha_{ij}^{k}\cdot {\left(W_{\text{att}}^{k}\right)}^{\left(1\right)}h_{j}^{\left(1\right)}\right) \end{array}$$

Here, σ denotes the activation function, *K* represents the number of attention heads, *N*_*i*_ signifies the neighborhood of node *i*, $\alpha_{ij}^{k}$ represents the attention coefficient for node *j* with respect to node *i* in the *k*-th attention head, $W_{\text{att}}^{k}$ is the weight matrix for attention in the *k*-th head, and $h_{j}^{\left(1\right)}$ denotes the feature vector of node *j* after the first graph convolutional layer.

Finally, inspired by the work of Wang et al. (2019) on node feature fusion, this study further designs a feature dual-fusion module, which considers both concatenation and element-wise product operations to integrate the node features outputted by each graph attention layer. We posit that the concatenation operation helps preserve more node feature information, while the element-wise product operation emphasizes the correlation between node features. We demonstrated the effectiveness of this fusion module in ablation experiments. The node features outputted by each graph attention layer in the GCAT feature encoder can be represented as {*Z*^(1)^, *Z*^(2)^, ⋯ , *Z*^(*N*)^}. Then, the feature dual-fusion module can be represented by the following equation:


(12) $$\begin{array}{l} \begin{array}{c} Z=\left(Z^{\left(1\right)}||Z^{\left(2\right)}||\cdots ||Z^{\left(N\right)}\right)W_{a}\\ +\left(Z^{\left(1\right)}⊙Z^{\left(2\right)}⊙\cdots ⊙Z^{\left(N\right)}\right)W_{b} \end{array} \end{array}$$

Here, || represents concatenation, and ⊙ represents element-wise (Hadamard) product, $W_{a}\in {\mathbb{R}}^{\left(N\times F\right)\times F}$ and $W_{b}\in {\mathbb{R}}^{F\times F}$ denote the trainable weight matrices, Z represents the final node feature.

In summary, this study represents the final microbe and disease features obtained from the microbe-disease association network as $Z_{A}^{m}\in {\mathbb{R}}^{n_{m}\times F}$ and $Z_{A}^{d}\in {\mathbb{R}}^{n_{d}\times F}$, respectively. Similarly, the microbe features obtained from the microbe similarity network are represented as $Z_{S}^{m}\in {\mathbb{R}}^{n_{m}\times F}$, and the disease features obtained from the disease similarity network are represented as $Z_{S}^{d}\in {\mathbb{R}}^{n_{d}\times F}$.

#### 2.3.3 Contrastive learning

Inspired by the work of Jin et al. (2024) on miRNA-disease association prediction, this study introduces contrastive learning to enhance the consistency of features of the same nodes across different association networks and the distinctiveness of features between different pairs of nodes. This approach leverages the complementary information among various association networks to obtain more effective representations of microbe and disease features. This module employs the contrastive loss function proposed by Zhu et al. (2020) for graph nodes. It considers the node features of the same disease *d*_*i*_ obtained from different association networks $\left(Z_{A}^{d_{i}},Z_{S}^{d_{i}}\right)$ as positive samples, while all other pairs of different nodes form negative sample pairs. Therefore, the contrastive learning loss function *Loss*_*d*_ for disease node features across different association networks can be expressed as:


(13) $$\begin{array}{l} \begin{array}{c} l\left(Z_{A}^{d_{i}},Z_{S}^{d_{i}}\right)=\\ \log\left(\frac{e^{\theta \left(Z_{A}^{d_{i}},Z_{S}^{d_{i}}\right)/\tau }}{e^{\theta \left(Z_{A}^{d_{i}},Z_{S}^{d_{i}}\right)/\tau }+\sum_{k\neq i}\left(e^{\theta \left(Z_{A}^{d_{i}},Z_{A}^{d_{k}}\right)/\tau }+e^{\theta \left(Z_{A}^{d_{i}},Z_{S}^{d_{k}}\right)/\tau }\right)}\right) \end{array} \end{array}$$

(14) $$\begin{array}{l} {\text{Loss}}_{d}=-\frac{1}{2n_{d}}\overset{n_{d}}{\underset{i=1}{\sum }}\left[l\left(Z_{A}^{d_{i}},Z_{S}^{d_{i}}\right)+l\left(Z_{S}^{d_{i}},Z_{A}^{d_{i}}\right)\right] \end{array}$$

where θ(·) is the cosine similarity, τ is a temperature parameter, *n*_*d*_ denotes the number of disease. Similarly, the contrastive learning loss function Loss_*m*_ for microbe node features across different association networks can be formulated as follows:


(15) $$\begin{array}{l} {\text{Loss}}_{m}=\frac{1}{2n_{m}}\overset{n_{m}}{\underset{i=1}{\sum }}\left[l\left(Z_{A}^{m_{i}},Z_{S}^{m_{i}}\right)+l\left(Z_{S}^{m_{i}},Z_{A}^{m_{i}}\right)\right] \end{array}$$

where *n*_*m*_ denotes the number of microbe. Therefore, the overall loss function of the GCATCMDA model in the contrastive learning module is formulated as follows:


(16) $$\begin{array}{l} {Loss}_{\text{contrast}}=\left({Loss}_{d}+{Loss}_{m}\right) \end{array}$$

#### 2.3.4 Microbe—disease associations prediction

This study aggregates the node features of microbes and diseases obtained from different association networks through vector concatenation, resulting in the final microbial feature representation $Z_{m}=\left[Z_{A}^{m}||Z_{S}^{m}\right]\in {\mathbb{R}}^{n_{m}\times 2F}$ and disease feature representation $Z_{d}=\left[Z_{A}^{d}||Z_{S}^{d}\right]\in {\mathbb{R}}^{n_{d}\times 2F}$. Subsequently, these aggregated feature representations are passed into an inner product decoder to compute the association scores between microbes and diseases. The calculation process is as follows:


(17) $$\begin{array}{l} A^{\prime }=\text{sigmoid}\left(Z_{m}Z_{d}^{T}\right) \end{array}$$

Where sigmoid is the activation function defined as 1/(1+*e*^−*x*^), which maps output values to the interval (0, 1), $A_{ij}^{\prime }$ represents the association prediction score between microbe *m*_*i*_ and disease *d*_*j*_.

Finally, the training of the GCATCMDA model employs Binary Cross-Entropy as the loss function for microbe-disease association prediction. The formula for this function is as follows:


(18) $$\begin{array}{l} {\text{Loss}}_{\text{classify}}=-\frac{1}{N}\sum_{\left(i,j)\in \{N^{+}\cup N^{-}\right\}}\left[A_{\left(i,j\right)}\log(A_{ij}^{\prime }\right)\\ +(1-A_{\left(i,j\right)})\log(1-A_{ij}^{\prime })] \end{array}$$

Where *N* denotes the total number of associations between microbes and diseases, *N*^+^ represents the confirmed associations between microbes and diseases, and *N*^−^ represents the associations yet to be confirmed. The tuple (*i, j*) represents the association between microbe *m*_*i*_ and disease *d*_*j*_. If (*m*_*i*_, *d*_*j*_) belongs to *N*^+^, then *A*_(*i, j*)_ = 1; otherwise, *A*_(*i, j*)_ = 0. $A_{ij}^{\prime }$ denotes the predicted association score by the model for this association pair. Therefore, the overall loss function of the GCATCMDA model can be expressed as:


(19) $$\begin{array}{l} Loss_{\text{total}}=Loss_{\text{classify}}+\lambda Loss_{\text{contrast}} \end{array}$$

Where λ represents the weighting parameter for the contrastive learning loss. The detailed steps of GCAT to predict novel microbe—disease associations is described in Algorithm 1.

**Algorithm 1:** GCAT framework for microbe-disease association.

1: Input: Microbe-disease associations **x**, real associations **y**
2: Output: Loss value
3: *x*_*micro*←Gaussion_kernel1(**x**)
4: *x*_*disease*←Gaussion_kernel2(**x**)
5: *micro*_*f*_*association, disease*_*f*_*association*←GCAT_association(**x**)
6: *micro*_*f*_*similarity*←GCAT_micro_similarity(*x*_*micro*)
7: *disease*_*f*_*similarity*←GCAT_disease_similarity(*x*_*disease*)
8: *micro*_*feature*←cat([*micro*_*f*_*association, micro*_*f*_*similarity*], dim = −1)
9: *disease*_*feature*←cat([*disease*_*f*_*association, disease*_*f*_*similarity*], dim = −1)
10: *pred*←Sigmoid(*micro*_feature*disease_*feature*)
11: *loss*←Binary_Cross_Entropy(*pred*, **y**)
12: *micro*_*contrastive*_*loss*←Contrastive_Loss(*micro*_*f*_*similarity, micro*_*f*_*similarity*)
13: *disease*_*contrastive*_*loss*←Contrastive_Loss(*disease*_*f*_*similarity, disease*_*f*_*association*)
14: *loss*←*loss* + *micro*_*contrastive*_*loss*+*disease*_*contrastive*_*loss*
15: return *loss*

## 3 Results and discussion

In this section, we will provide an exposition of the experimental setup and subsequently delve into an analysis and discussion of the experimental results.

### 3.1 Experimental setup

The GCATCMDA model proposed in this study is a microbe-disease association prediction model based on graph neural networks and contrastive learning. It aims to predict potential associations between microbes and diseases from known microbial-disease association dataset. The hyperparameter settings required for this model are described as follows. Firstly, the Gaussian kernel similarity threshold *t* needs to be set for constructing microbe and disease similarity networks. Secondly, parameters need to be set for the GCAT feature encoder module, including the dimensionality *F* of node features, the number of network layers *L* for graph convolution, and the number of attention heads *heads* for the graph attention mechanism. Then, in the contrastive learning loss module, the temperature hyperparameter τ and the weight parameter λ relative to the total loss are adjusted. Finally, the GCATCMDA model is trained using the Adam (Kingma and Ba, 2014) optimizer, with parameters including the learning rate *lr*, weight decay *wd*, and the number of training iterations *epochs*.

This study determines the optimal parameter settings of the GCATCMDA model on the dataset by enumerating different parameter combinations. Subsequently, there is an analysis of key parameters *t*, *F*, *L*, and *heads*. After comparing experimental results, the optimal hyperparameter settings for the GCATCMDA model on the HMDAD dataset are determined as follows: *t* = 0.4, *F* = 128, *L* = 3, *heads* = 2, τ = 1, λ = 0.2, *lr* = 0.00001, *wd* = 0.001, and *epochs* = 100.

In order to verify the effectiveness of the proposed GCATCMDA model, we compares it with KATZHMDA (Chen et al., 2017), LRLSHMDA (Wang et al., 2017), NTSHMDA (Luo and Long, 2018), and KGNMDA (Jiang et al., 2022). These five methods are recognized for their outstanding performance in this task in past studies and provide research methods with open-source code. For negative samples required in model training, this study randomly selects an equal number of negative samples from all unknown microbe-disease association pairs. The number of negative samples matched the number of positive samples as to maintain a balanced dataset. In each cross-validation experiment, the Gaussian kernel similarity scores for microbes and diseases are recalculated based on the training set to ensure the effectiveness of evaluating model performance through the test set. In this experiment, we employ the same dataset and follow the hyperparameter settings used in the original papers or provide open source codes for other compared models. We adopted the same evaluation metrics as the previous study (Jiang et al., 2022), including the area under the ROC curve (AUC) and the area under the precision-recall curve (AUPR) to assess the performance of the models. To evaluate the performance of these models in predicting potential associations between microbes and diseases, this study conducted 10 repetitions of five-fold cross-validation experiments and 10 repetitions of ten-fold cross-validation experiments by setting different random seeds, and then computed the average to ensure the accuracy of our results.

### 3.2 The classification performance of models

**Figure 3:**
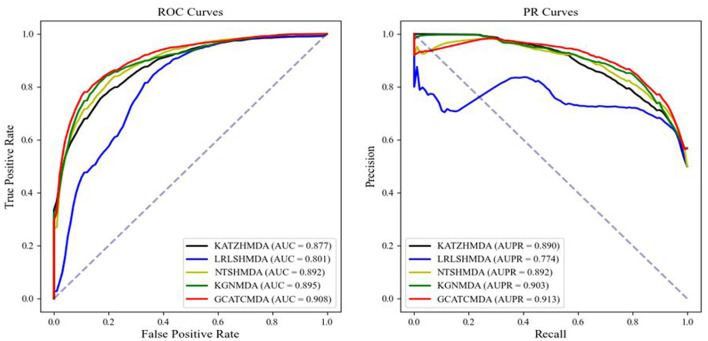
The AUROC curve and AUPR curve of five-fold CV on the HMDAD datasets between different methods.

**Figure 4:**
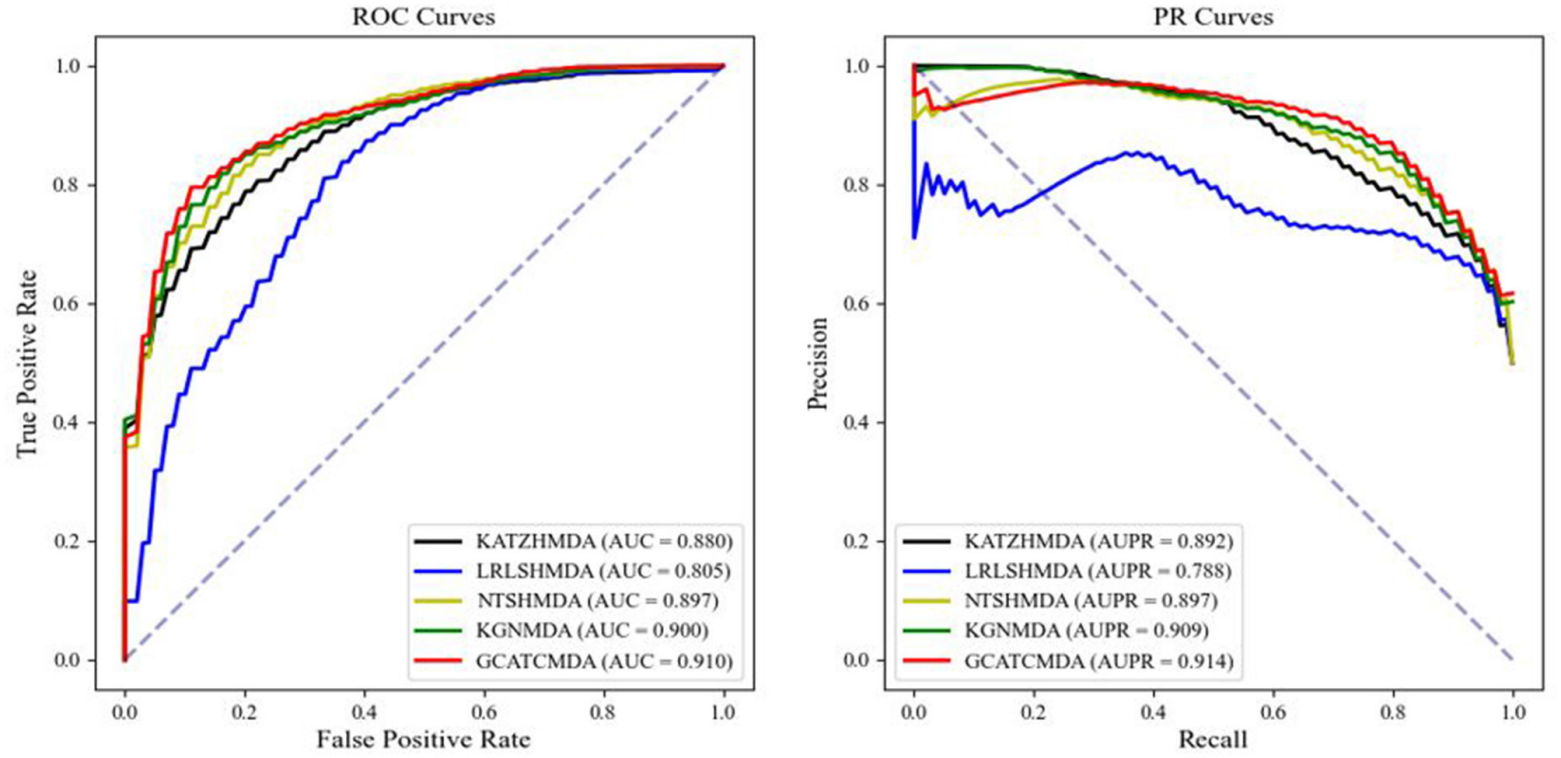
The AUROC curve and AUPR curve of 10-fold CV on the HMDAD datasets between different methods.

The comparative results of the two cross-validation experiments conducted on the HMDAD dataset for the five aforementioned models are presented in Table 1. The optimal performance is highlighted in bold, with standard deviations indicated in parentheses. To provide readers with a clearer visualization of the performance of the models, this study further plotted the ROC curve and PR curve, as shown in Figures 3, 4, respectively.

**Table 1:** Classification performance comparison of GCATCMDA with existing methods.

**Cross-validation**	**Methods**	**AUC**	**AUPR**
Five-fold-CV	KATZHMDA	0.877 (0.023)	0.890 (0.021)
	LRLSHMDA	0.801 (0.032)	0.774 (0.039)
	NTSHMDA	0.892 (0.028)	0.892 (0.036)
	KGNMDA	0.895 (0.021)	0.903 (0.020)
	GCATCMDA	**0.908 (0.020)**	**0.913 (0.022)**
10-fold-CV	KATZHMDA	0.880 (0.031)	0.892 (0.027)
	LRLSHMDA	0.805 (0.047)	0.788 (0.058)
	NTSHMDA	0.897 (0.030)	0.897 (0.038)
	KGNMDA	0.900 (0.029)	0.909 (0.029)
	GCATCMDA	**0.910 (0.026)**	**0.914 (0.033)**

The values in bold represent the best ones.

From the experimental data presented above, it can be observed that the GCATCMDA model proposed in this study has achieved excellent predictive performance in the task of predicting associations between microbes and diseases, surpassing methods proposed in previous studies. For instance, in the five-fold cross-validation experiment, the model obtained an approximate 1.3% improvement in AUC compared to the best previous predictive performance. Similarly, in the ten-fold cross-validation experiment, the model obtained an approximate 1.0% enhancement in AUC compared to the best previous predictive performance. The improvement in predictive performance was slightly more pronounced in the fold-fold cross-validation compared to the 10-fold cross-validation. This can be attributed to the larger training sets used in the 10-fold validation, which reduce variability across folds and provide more comprehensive data for model training. However, the reduced variability can lead to subtler improvements in performance metrics, as the model benefits from a more stable but less varied dataset. In contrast, the five-fold validation, with its larger test sets, introduces more variability, making performance improvements more apparent.

Graph transformer models offer strong capabilities in capturing global node features through their self-attention mechanisms (Li et al., 2024a,b). This allows them to handle complex and non-local structures, which can be beneficial for highly heterogeneous datasets. However, these models come with significant computational complexity, scaling quadratically with the number of nodes, making them less practical for large datasets like microbe-disease networks.

While the GCATCMDA model combines GCN and GAT to effectively capture both local features and selective attention on relevant neighbors, Graph Transformer models are designed to capture these relationships on a broader scale. The full attention mechanism of Graph Transformers allows them to dynamically weigh the importance of distant nodes, offering more flexibility in feature extraction across large and complex networks. In contrast, our GCATCMDA model, which combines GCNs and GATs, is more computationally efficient and particularly suited to smaller, sparser datasets like the HMDAD database. While graph transformers excel in capturing global relationships, our approach balances local feature aggregation and attention, offering a more efficient solution. Future work could explore integrating graph transformers to leverage their global feature-capturing capabilities alongside our model's efficiency in handling localized data.

### 3.3 Parameter analysis

The GCATCMDA model proposed in this study possesses several crucial parameters, such as the Gaussian kernel similarity threshold *t* for constructing microbe and disease similarity networks, the dimensionality *F* of node features, the number of network layers *L* for graph convolution, and the number of attention heads *heads* for the graph attention mechanism. Therefore, this study conducted training with different parameter combinations on the HMDAD dataset and utilized the experimental results from 10 repetitions of five-fold cross-validation to analyze the impact of these parameters on the model's performance.

**Figure 5:**
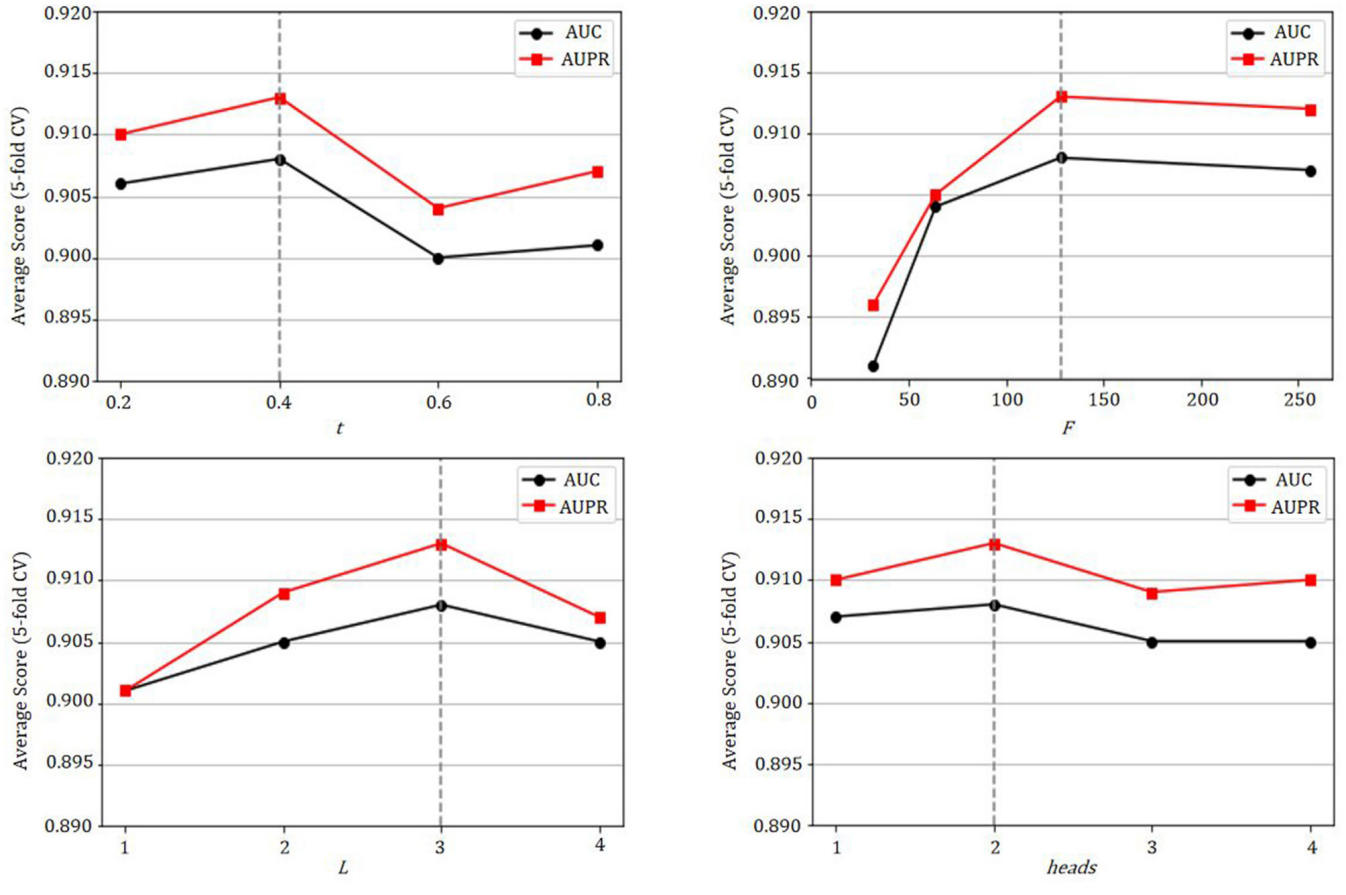
The effect of parameters *t*, *F*, *L*, and *heads* on the GCATCMDA model.

As shown in Figure 5, the model fails to achieve the best predictive performance when the Gaussian kernel similarity threshold *t* is either set too high or too low, the optimal predictive performance of the model is attained when *t* = 0.4. Moreover, as the dimensionality of node features increases, the predictive performance of the model gradually improves, with the best performance observed when *F* = 128. Additionally, the model exhibits its best predictive performance when the number of network layers for graph convolution *L* = 3. Furthermore, it is observed that the evaluation metrics AUC and AUPR attain their maximum values when the number of attention heads for the graph attention mechanism *heads* = 2.

### 3.4 Ablation studies

**Figure 6:**
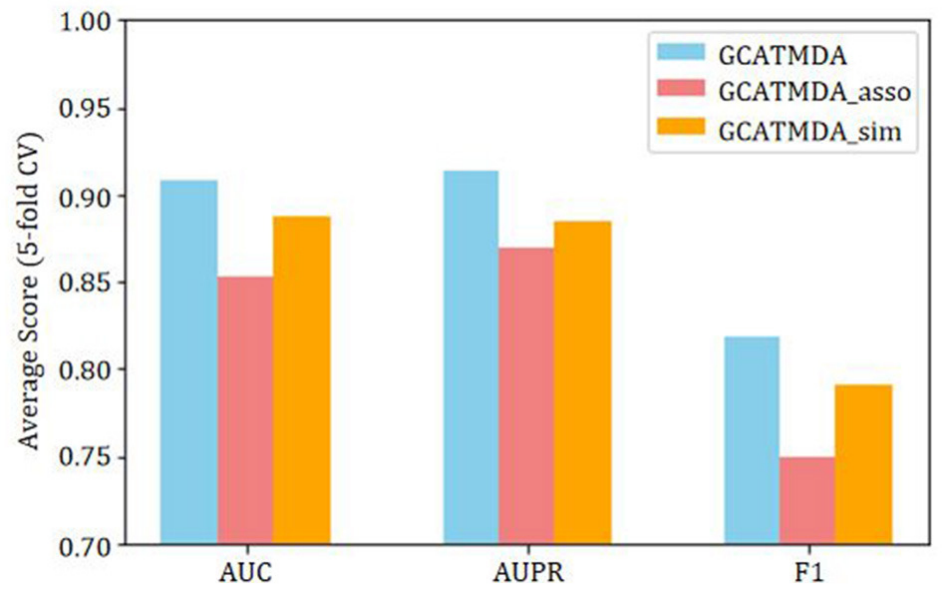
Effect of node embedding extracted from different networks on prediction.

To further validate the impact of each module in the GCATCMDA model on the prediction performance of microbe-disease associations, this study conducted ablation experiments on the HMDAD dataset. The evaluation metrics included AUC, AUPR, Precision, Recall, and F1 score. These metrics aimed to comprehensively analyze the influence of different modules on the performance of the GCATCMDA model. The experimental results represent the average scores of 10 repetitions of five-fold cross-validation experiments. Initially, given that the GCATCMDA model simultaneously utilizes microbe similarity networks, disease similarity networks, and microbe-disease association networks to learn the feature representations of microbes and diseases, this study assessed the impact of node features from different association networks on the model's prediction performance. The experimental results are illustrated in Figure 6, where GCATCMDA_sim denotes learning the feature representations of microbes and diseases only from microbe and disease similarity networks, while GCATCMDA_asso denotes learning the feature representations only from the microbe-disease association network. It can be observed from Figure 6 that integrating feature representations of microbes and diseases from different association networks effectively enhances the model's predictive performance.

Next, given that the GCATCMDA model mainly consists of GCN, GAT, feature dual fusion module, and contrastive learning module, this study attempted to remove each module individually to investigate the impact of different modules on the model's prediction performance. The experimental results are presented in Table 2, where “GCATCMDA_GCN” denotes the removal of the graph convolutional network from the original model, “GCATCMDA_GAT” denotes the removal of the graph attention mechanism, “GCATCMDA_SUM” denotes replacing the feature dual fusion module of the original model with a simple summation operation, and “GCATCMDA_CL” denotes the removal of the contrastive learning module from the original model. From the results in Table 2, it can be observed that both “GCATCMDA_GCN” and “GCATCMDA_GAT” exhibit lower predictive performance compared to the original model, indicating that the integration of graph convolutional networks and graph attention mechanisms for node feature learning is effective in obtaining more informative node feature representations from the network. The predictive performance of “GCATCMDA_SUM” is also lower than that of the original model, suggesting that the designed feature dual fusion module effectively fuses node feature information outputted by the graph attention layers. Similarly, the predictive performance of “GCATCMDA_CL” is slightly lower than that of the original model, indicating that the addition of the contrastive learning module can improve the model's predictive performance to some extent.

**Table 2:** Classification performance comparison of GCATCMDA with existing methods.

**Method**	**AUC**	**AUPR**	**Precision**	**Recall**	**F1**
GCATCMDA_GCN	0.893 (0.023)	0.903 (0.021)	0.858 (0.033)	0.771 (0.041)	0.812 (0.027)
GCATCMDA_GAT	0.884 (0.034)	0.900 (0.033)	0.865 (0.040)	0.737 (0.086)	0.793 (0.062)
GCATCMDA_SUM	0.894 (0.028)	0.887 (0.040)	0.866 (0.045)	0.749 (0.078)	0.801 (0.056)
GCATCMDA_CL	0.904 (0.021)	0.908 (0.025)	0.869 (0.034)	0.770 (0.039)	0.816 (0.028)
GCATCMDA	**0.908 (0.020)**	**0.913 (0.022)**	**0.874 (0.034)**	**0.772 (0.045)**	**0.819 (0.032)**

The values in bold represent the best ones.

**Figure 7:**
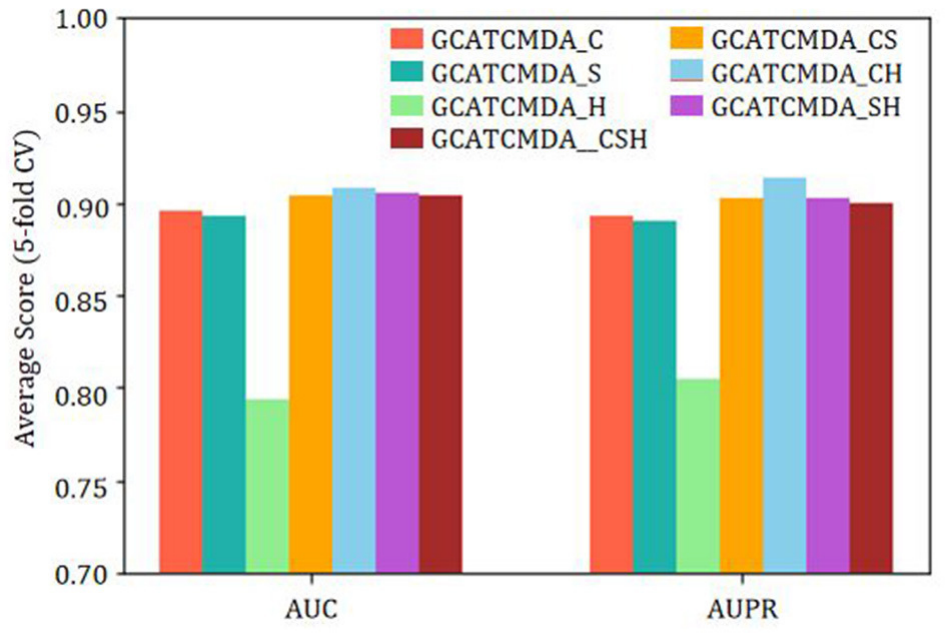
The impact of different feature fusion methods on model prediction performance.

Finally, to investigate the impact of different operations for fusing node features outputted by the graph attention layers on the GCATCMDA model prediction performance, this study sophisticatedly combined three common feature vector operations: concatenation, sum, and element-wise product. The combined fusion feature formulas are similar to the feature dual fusion formula described earlier. The experimental results are illustrated in Figure 7. GCATCMDA_C represents the use of concatenation only, GCATCMDA_S represents the use of sum only, and GCATCMDA_H represents the use of element-wise product only. GCATCMDA_CS represents the combination of concatenation and sum, GCATCMDA_CH represents the combination of concatenation and element-wise product, GCATCMDA_SH represents the combination of sum and element-wise product, and GCATCMDA_CSH represents the combination of concatenation, sum, and element-wise product. From the experimental results in Figure 7, it can be observed that selecting the combination operations of concatenation and element-wise product in the feature dual fusion module can most effectively fuse node features outputted by the graph attention layers.

### 3.5 Case studies

To further validate whether the GCATCMDA model can predict associations between microbes and diseases, this study initially trained the model using all known microbial-disease associations in the HMDAD dataset. Subsequently, obesity and inflammatory bowel disease (IBD), two common diseases, were selected as subjects for case analysis. The model predicted microbial associations with obesity and IBD by sorting the predicted association scores from high to low and retaining the top 20 unknown microbial associations with high scores for these two diseases. Finally, employing a literature search approach, this study validated whether these microbial associations with diseases existed by examining relevant publications in the biomedical literature database PubMed. This validation process aimed to assess the accuracy of the microbial-disease associations predicted by the GCATCMDA model.

From Table 3, it can be observed that among the top 20 associated microbes identified by the GCATCMDA model for obesity, 16 of them have been previously documented in the literature to be associated with obesity. For instance, Xu et al. (2022), by reviewing literature on gut microbiota and obesity, identified an association between Prevotella and obesity. Baradaran et al. (2021) experimentally demonstrated that individuals positive for *Helicobacter pylori* infection are more likely to suffer from obesity, with an increased risk of *Helicobacter pylori* infection among obese individuals. From Table 4, it can be observed that in IBD, among the top 20 associated microbes identified by the GCATCMDA model, 15 have been previously demonstrated to be associated with IBD in the literature. For example, Quaglio et al. (2022) demonstrated that the abundance of Bacteroidetes and Firmicutes in patients with IBD undergoes significant changes. Cardoneanu et al. (2021) experimental research showed a significant decrease in the abundance of Clostridium coccoides in patients with IBD compared to healthy individuals.

**Table 3:** Candidate microbes related to obesity predicted by GCATCMDA model.

**Rank**	**Microbe**	**Evidence**	**Rank**	**Microbe**	**Evidence**
1	*Prevotella*	PMID:35093025	11	*Enterobacter aerogenes*	Unconfirmed
2	Proteobacteria	PMID:31197613	12	*Enterobacter hormaechei*	Unconfirmed
3	*Helicobacter pylori*	PMID:34243821	13	*Klebsiella pneumoniae*	PMID:31921729
4	Lachnospiraceae	PMID:31397240	14	*Shigella dysenteriae*	Unconfirmed
5	Actinobacteria	PMID:19043404	15	*Haemophilus*	PMID:31976177
6	*Staphylococcus*	PMID:29667480	16	*Clostridium coccoides*	PMID:29667480
7	*Enterococcus*	PMID:35967777	17	*Betaproteobacteria*	Unconfirmed
8	*Clostridium*	PMID:29667480	18	*Clostridium leptum*	PMID:36756620
9	*Clostridium difficile*	PMID:25638400	19	Bacteroidales	PMID:33407104
10	*Faecalibacterium prausnitzii*	PMID:23985870	20	*Enterococcus faecium*	PMID:36590404

**Table 4:** Candidate microbes related to IBD predicted by GCATCMDA model.

**Rank**	**Microbe**	**Evidence**	**Rank**	**Microbe**	**Evidence**
1	Bacteroidetes	PMID:36157114	11	*Enterobacter hormaechei*	Unconfirmed
2	Firmicutes	PMID:36157114	12	*Klebsiella pneumoniae*	PMID:36436756
3	*Clostridium coccoides*	PMID:33548121	13	*Shigella dysenteriae*	Unconfirmed
4	*Helicobacter pylori*	PMID:30237392	14	*Clostridium leptum*	PMID:33548121
5	*Prevotella*	PMID:38053528	15	Lysobacter	Unconfirmed
6	*Clostridium difficile*	PMID:31698044	16	Rickettsiales	Unconfirmed
7	*Staphylococcus*	PMID:31662859	17	*Streptococcus mitis*	PMID:30796823
8	*Staphylococcus aureus*	PMID:31698044	18	*Xanthomonas*	PMID:35689701
9	*Enterococcus*	PMID:32292819	19	Enterobacteriaceae	PMID:24629344
10	*Enterobacter aerogenes*	Unconfirmed	20	*Lactobacillus*	PMID:37773196

In summary, it can be observed from Tables 3, 4 that the GCATCMDA model achieves an accuracy of over 75% in predicting potential associated microbes for both obesity and inflammatory bowel disease. Therefore, this study concludes that the GCATCMDA model can provide effective and accurate candidate sets of microbes associated with diseases, thereby reducing the research costs and duration of traditional biological experiments.

## 4 Conclusion

This article primarily introduces the GCATCMDA model proposed in this study, aimed at predicting potential sets of microbe-disease associations based on known microbe-disease association data. Initially, the article outlines the construction of Gaussian kernel similarity networks for microbes and diseases using known association data and explains how the model combines graph neural networks with contrastive learning to obtain effective feature representations for microbes and diseases. Subsequently, experimental evaluations are conducted to compare the GCATCMDA model with existing methods, demonstrating its superiority in microbe-disease association prediction tasks. Additionally, parameter analysis experiments validate the rationality of parameter settings in the GCATCMDA model, while ablation experiments confirm the effectiveness of each module in the model. Finally, obesity and inflammatory bowel disease are selected as case studies to validate the high accuracy of the microbe-disease association candidate sets predicted by the GCATCMDA model.

The proposed model combines GCN and GAT to leverage the strengths of both approaches. GCN effectively captures local neighborhood information by performing convolution operations over graph structures, allowing the model to aggregate features across connected nodes. However, GCN applies equal weighting to all neighboring nodes, which may limit its ability to differentiate between more and less important neighbors. To address this limitation, GAT introduces an attention mechanism that assigns different importance to neighboring nodes by computing attention coefficients. This allows the model to focus more on the relevant nodes, improving its ability to capture complex interactions. By combining GCN's ability to aggregate global structural information with GAT's selective attention on important neighbors, the proposed model effectively captures both local and global patterns within the graph, leading to enhanced predictive performance.

While our study has demonstrated the effectiveness of the GCATCMDA model in predicting microbe-disease associations, there are several limitations that must be acknowledged. First, the model has only been evaluated using the HMDAD database, and its generalization ability requires further validation across other public datasets, such as HMDA and Disbiome. The limited volume of data in this study may also hinder the model's ability to capture complex patterns, suggesting the need for more extensive datasets to enhance its predictive performance. Additionally, our current approach does not differentiate between positive and negative association information, a distinction that will be addressed in future research to refine prediction accuracy. By overcoming these limitations, we anticipate further improvements in the model's robustness and its potential application across a broader range of microbial and disease studies.

In conclusion, this study asserts that the GCATCMDA model can advance the development of deep learning algorithms in the field of microbe-disease association prediction. Moreover, it effectively aids biologists in exploring potential associations between microbes and human diseases from a big data perspective, thereby reducing the costs of traditional biological experiments and accelerating research progress in the field of gut microbes and disease association studies.

## Data Availability

The HMDAD database is available at: http://www.cuilab.cn/hmdad. The source code is available upon reasonable request to the corresponding authors.
